# "The Hospital" Nursing Mirror

**Published:** 1900-03-24

**Authors:** 


"The Hospital" Nursing Mirror.

Being the Nursing Section of "The Hospital."

[Contributions for this Section of "The Hospital" should be addressed to the Editor, The Hospital, 28 & 29, Southampton Street, Strand, London, W.C., and should have the word "Nursing" plainly written in left-hand top corner of the envelope.]

### Notes on News from the Nursing World.

### THE ROYAL RED CROSS FOR A PRINCESS.

The Queen has conferred the much-valued decoration of the Royal Red Cross upon Princess Victoria of Schleswig-Holstein. In this case the dark blue ribbon with red edge has been given by her Majesty to her grand-daughter for zeal and devotion in providing for the sick and wounded soldiers. This honour is partly due to the interest that the Princess has taken in starting, in conjunction with many other influential persons, the Children's Penny Fund, by means of which children of all sorts and conditions, in towns and villages, in homes and schools, contribute their pence for the benefit of the convalescent soldiers, and partly to the solicitude she has shown the patients at Netley. The pretty little anecdote about the Princess and the quilt, told in our columns a fortnight ago, will be fresh in the memory of our readers. Amongst other Royal possessors of the Red Cross are the Queen herself, the Princess of Wales, the Empress Frederick, the Queen of Greece, and the Duchess of Sparta.

### THE "PRINCESS OF WALES" HOSPITAL SHIP.

The inaccuracy of the statement that the hospital ship "Princess of Wales" would not return to South Africa has been conclusively proved by the fact that the vessel is about to leave Southampton for the Cape, much to the delight of the nurses. It will be remembered that the Princess of Wales despatched some little memento for each of the nurses on board, but it appears that up to the time of their departure to England with their first party of patients they had not received the gifts. They hope on their arrival at their destination to find the parcels awaiting them. Unless it is found necessary to hurry back again with wounded soldiers the ship is likely to remain for a short time in South African waters. The Princess of Wales has considerately forwarded a large quantity of warm clothing and other things designed to promote the comfort and welfare of the prospective soldier patients who may be expected to be sent on board soon after the arrival of the vessel at the Cape.

### THE NURSES OF WESTERN AUSTRALIA AND THE WAR.

The Matron of the Perth Public Hospital, who belongs to the Imperial Nursing Corps, and has been ordered to the front, confirms the statement that the Colony can ill afford to spare any of its trained nurses, but she agrees with other people that the experience gained by those who have been selected to tend the Colonial troops will find their experience of much value in after life. In fact, she "considers that it is impossible to over estimate the advantage it will be to them." Miss Wemyss describes herself as "born in the army." Her father was the commander of a regiment of Hussars, the late General Wauchope was her cousin, and Colonel Stafford, who was killed near the Modder River, was also a relation. She gives up her appointment at Perth with regret. Though under ordinary circumstances she would gladly have remained there, she naturally welcomes the call for service in South Africa. As several correspondents have written to us about nursing in Western Australia, we advise them to apply for information to the Agent-General for the Colony, Westminster Chambers, 1, Victoria Street, S.W.

### THE REFUGEES' MATERNITY HOME AT DURBAN.

It having been found necessary by the Ladies' Relief Committee to start a maternity home, the wives of three doctors undertook to act upon the committee. Two thousand pounds were collected to start the institution, and furniture was given by generous friends. Lately the Mansion House Fund has contributed a weekly sum for the support of the home, local funds being exhausted. The home consists of a small cottage, with a matron and one nurse, and there are so many cases requiring admission that, as soon as it is at all feasible, the mothers are discharged so as to make room for others. The ladies of Durban are most kind in helping to the utmost of their power. Those who live near the little hospital take it in turns to send beef tea, puddings, milk, &c., each family arranging to be responsible one day in the week. Frequently, if the baby or parent is very delicate, some good Samaritan receives the pair as guests for a week or two, or makes them a private present to buy additional nourishing food. The shilling a day allowed to refugees by the Mansion House Fund naturally permits of nothing but real necessities being purchased.

### A BRITISH NURSE IN PRETORIA.

It is not only British nurses in Johannesburg who have been badly treated by the Boers. The only nurse in Pretoria at the time the war broke out who was a British subject states that for four or five weeks afterwards she went on happily with her work. But the morning after Mr. Winston Churchill's escape was discovered she was visited by a big bully of a field cornet, who had been given the power to turn her out of the Red Cross Hospital to which she was attached. She says:—

"He was evidently under the impression that he was speaking to one of his own Boer women, as he shouted at me in the corridor until I invited him into the sitting-room. He informed me that I could not be trusted, because how could they tell that I was not carrying information to the officers or passing letters to them, and I would, therefore, 'be turned out' of the Red Cross. I accepted my dismissal with a bow, but took no notice of it, as I thought he was bullying me and the matter would blow over. I continued my work as usual until eight that night, when I went for a walk. On passing the hospital a little later on I saw five armed men on horseback at the door, and when I returned later I was greeted with the news that the police had been there to search for Mr. Churchill and turn me out at the same time. Permission was eventually given that I might stay till the next morning, but on no condition was I to go near my patients again. A friend of mine living close by heard of what had happened, and sent for me to stay with her until I should hear of a boat to take me from Lourenço Marques to Durban. Most of the Pretorians believed that I had aided

Mr. Churchill. It was quite interesting to hear what I was supposed to have done to help him. He is described as about my size, and therefore I lent him one of my nursing costumes, and later on I heard I had also given him my permit. I had no chance to clear myself, as martial law had been proclaimed, and I simply had to obey orders and go."

### THE ADELAIDE WAR NURSES.

The six nurses who were selected out of 26 applicants for service in South Africa have by now reached the seat of the war. Their fares both ways are to be paid, they receive a sum down for equipment, they are given a salary of 15s. a week while on duty, and the amount realised by the sale of their photographs, taken free of charge by a local photographer, is to be placed to their credit. The weekly payment is unusual in this country, and under some circumstances might be an unsatisfactory arrangement, whilst the artist has decidedly chosen a somewhat novel manner of showing his appreciation of the labours of the nursing profession.

### THE ROYAL NATIONAL PENSION FUND FOR NURSES.

At the thirteenth annual meeting of the Royal National Pension Fund for Nurses, which is fully reported elsewhere, Sir Henry Burdett, who moved the adoption of the report, showing a large increase in the number of proposals and of policies issued, pointed out that a remarkable feature of the year's work was that the withdrawals had been less than in 1898. Taking the average of three years, Sir Henry observed, it was found that of the total number of withdrawals about 20 per cent. were due to the holders giving up nursing as a vocation, and about the same percentage to the fact that they were contemplating marriage. The number of annuitants was 223. In the sickness branch the sum of £1,297 was distributed as sickness allowance. Sir William Broadbent, who seconded the adoption of the report, dwelt on the importance of the sick fund, and said that nothing but the best business capacity could have brought the Society to its present position. An interesting feature of the proceedings was the vote of thanks to Mr. Dick, the secretary of the Fund, whose success in organising the reception of 1,200 nurses at Marlborough House in the summer has been warmly recognised by the members of the nursing profession.

### NURSING IN IRISH WORKHOUSES.

Irish workhouse reform is in the air, and at a recent meeting of the Irish Workhouse Reform Association at Dublin various proposals for improvements in the existing system were made. A resolution to the effect that workhouses should be converted into district hospitals, and that auxiliary asylums should be established outside workhouses for the care of epileptics, was adopted. With regard to nursing, as in English workhouse infirmaries, much remains to be done; but at least, thanks to the efforts of the association, pauper nursing in union hospitals is now practically a thing of the past. A satisfactory feature of the change is the demand thus created for trained nurses, and we observe in this connection that a plea is put forward by an Irish paper for Government assistance in the training of nurses. This demand is probably suggested by the fact that the supply of trained nurses seems unequal to the requirements of workhouse hospitals, but the supply depends upon the payment and other rewards of service, and the supply may be increased if the Irish Boards of Guardians offer sufficiently liberal salaries and make the conditions of work all that could be desired. At present the salaries are slender, and the conditions of work are often exceedingly defective. This also applies to the conditions under which nurses employed by English Poor Law Guardians work, and we suspect that the proposal for the training of nurses for workhouse infirmaries generally at the cost of the State is merely an attempt to obtain cheap nurses which would not meet with public support.

### THE NURSES' HOME AT BRISTOL ROYAL INFIRMARY.

The new wing of the Bristol Royal Infirmary Nurses' Home is now in use, and from the excellent arrangements made it is evident that the committee are determined to spare no effort to make their nursing staff thoroughly comfortable. The older part of the Home has been repapered and painted, and new furniture has been provided. The rooms on the south side are coloured a pretty shade of green, with which the carpet and tiles of washstands harmonise. Those on the north side are coloured terra-cotta. Each room contains a corner wardrobe with looking-glass panel, washstand with marble top and tiled back, writing-table, chest of drawers, two chairs, and a comfortable spring bedstead. Thorough ventilation is arranged for, in addition to the window, by an exit for foul air and another inlet for fresh air. The whole Home is lit by electricity. The sitting-room is a bright, cosily-furnished room, overlooking the garden. The library, or quiet room for writing and studying, is thoroughly comfortable, and suited for its purpose. Several small writing tables are provided, and a good library is being formed. The Home contains accommodation for 93 nurses. The garden is large enough to allow for a full-sized tennis-court, besides a good space to spare for walks and garden chairs.

### THE HAGGERSTON AND HOXTON NURSES.

The tenth annual meeting of the Haggerston and Hoxton District Nursing Association was held at 21, Carlton House Terrace, on Tuesday afternoon. Lady Frederick Cavendish presided in the absence through indisposition of Princess Christian, and the Bishop of Stepney was among the speakers present. The report shows a satisfactory state of progress. The association have acquired a third house, and the landlord has given permission for the erection of a large dining-room and four extra bed-rooms. It is hoped that the nurses will be settled in their enlarged quarters before the autumn. There will then be accommodation for a staff of 14, a limit which it is considered desirable not to exceed in one centre. During 1899 the nurses attended 1,551 patients, and paid 36,066 visits; some of the cases being very sad. The work is managed economically, the cost of each nursing visit being about 5½d., which, it is truly urged, is not much in comparison with the benefits conferred. The cost of the enlargement and furnishing of the home will be about £800, of which only half is as yet promised. It is therefore necessary to appeal to the public to contribute the remainder, and the cause is so good that we have no hesitation in recommending it to support.

### MORE NIGHT NURSES WANTED IN WORKHOUSE INFIRMARIES.

In the annual nursing return for the workhouses in Lancashire, Westmoreland, part of Cumberland, and part of the West Riding of Yorkshire, Mr. H. Jenner-Fust, jun., reports with satisfaction the continued decrease of the number of patients per nurse. Six years ago the figures were 17·48, and they are now 12·79. But there is still, it appears, a great need in several instances for more night nurses, and Mr. Jenner-Fust intimates his opinion that a further lessening of the number of paupers employed under the name of attendants is much to be desired. Obviously, the best way of bringing about this result would be to increase the staff of night nurses, and we hope that the Local Government Board will not only observe, but act upon, the report in reference to the important district under the charge of Mr. Jenner-Fust.

### THE STAGE NURSE.

The curious want of knowledge among the general public as to the regulations in force at well-managed hospitals is shown in the recognition given to what anyone who understands real nursing must regard as an objectionable and senseless farce just produced at a London theatre. The author of the play makes a nurse the principal performer, and depicts a probationer from "St. Benedict's" commissioned by a doctor to attend upon a young bachelor who is said to be dangerously ill. The fact that the illness of the fast young man is only invented by the physician to keep him out of harm's way, and that the nurse is a rich young heiress whom his friends are anxious that he should marry, is no excuse for the theory that the matron of a nursing home attached to a hospital is in the habit of sending out probationers to nurse young men in chambers. The dramatic critic who describes Nurse Finch as "quite a study of the type" is evidently quite unacquainted with the genuine nurse, whose characteristics are certainly not "an opulent sense of humour and coquetry," and who would not care to have herself described as "knowing enough, in somewhat unpleasant fashion."

### A NURSING ASSOCIATION IN EVERY VILLAGE.

At the sixth annual meeting of the Hucknall District Nursing Association Mr. Jesse Hind, in moving the adoption of the report, said "he trusted ere long to see a nursing association established in every village in the county." Mr. Hind is, of course, specially interested in Nottinghamshire, but in the not far distant future we hope to see a nursing association established in every village in the country. Of course, there will always be villages too small to need the individual attention of a nurse, but given the existence of an organisation and the necessary funds, there is no reason why eventually every village, or group of villages, should not have its trained nurse. At Hucknall a delegate of the pit club observed that he had watched the work of the nurses critically, and knew of several lives that were saved by them last year. He added, "If one life only had been saved the existence of the association would have been justified." It is with the view of saving life, as well as for the purpose of alleviating pain and restoring strength to suffering humanity that we want every village to possess a nurse of its own.

### THE BOLTON NURSES.

It is interesting to compare the eleventh annual report of the Bolton District Nursing Association with the first document of the kind. In 1889 the number of cases occupying the attention of the nurses was 303, necessitating 2,762 visits. In the year 1899 the cases were 1,475, and the visits reached a total of 33,461. During eight months the nursing staff consisted of the superintendent and nurses, but typhoid fever becoming prevalent towards the close of the year the committee were obliged to engage a tenth nurse. Typhoid has ceased to be prevalent, but it has been found essential to retain the services of the extra nurse, the cases attended lately varying from 110 to 120. In Bolton, as elsewhere, it appears that the artisan and other classes who benefit greatly by the association do not make any adequate return; and the committee make a strong appeal to them to contribute systematically to the support of an organisation which has done so much to brighten the lives and the homes of the inhabitants of the dark courts and alleys of the town.

### RESIGNATION OF A SISTER AT THE ROYAL INFIRMARY, LIVERPOOL.

The resignation from the nursing staff of the Liverpool Royal Infirmary of Sister Schröder, who has for many years most ably and faithfully discharged the duties of sister in charge of Dr. Glynn's female ward and the private wards of the institution, is deeply regretted. A Liverpool correspondent writes, "As a highly efficient and devoted nurse she will be missed by the honorary medical officers. As a bright and genial companion her loss is deplored by her co-workers; and as a friend she will always be remembered with sentiments of the utmost regard by every official of the hospital, and by a multitude of patients, who owe their present well-being to her unremitting care. The only consolation left her very large circle of friends is the fact that her desire has been gratified by her admission to the Army Nursing Service Reserve. That she may be spared health and strength to continue for many years in the good work nature qualified her to fulfil is the sincere and earnest wish of all who have the felicity to know her. On the eve of her departure for London the sisters of the hospital presented her with a very handsome jewel case, and the nurses' farewell gift consisted of a chatelaine fitted with the usual instruments."

### SHORT ITEMS.

A grand afternoon concert, promoted by the Ladies' Association, will be given on Tuesday, May 22nd, at three p.m., at the Queen's Hall, in aid of the Metropolitan Hospital, Kingsland Road, N.E., under the immediate patronage of Princess Louise (Marchioness of Lorne).—The American hospital ship "Maine" will arrive home in the middle of April, and will return to South Africa for further service immediately. There are on board 12 officers and 153 non-commissioned officers and men.—Nurse McCaul is a passenger for England in the "Dunvegan Castle," which also has Mr. Treves on board.—At the next entertainment at the National Hospital for the Paralysed and Epileptic, Queen Square, Bloomsbury, to be given on Thursday, the programme will be provided by Mr. Burford Rawlings.—We are requested to intimate that the reception to nurses announced by Bovril (Limited) in our advertising columns has had to be temporarily postponed, and that further particulars will be published later.

### Lectures on Medicine to Nurses.

By H. A. LATIMER, M.D. (Dunelm), M.R.C.S. Eng., L.S.A.Lond.; Consulting Surgeon, Swansea Hospital; Past President of the South Wales and Monmouthshire Branch of Brit. Med. Assoc.; Past President of the Swansea Medical Society; Hon. Life Member of the St. John Ambulance Association, &c.

### LECTURE IV.

FUNCTIONAL AND ORGANIC DISEASE (*continued*)—DIAGNOSIS, HEREDITY, AND TEMPERAMENTS.

IT results from the very nature of things and from the imperfections of ourselves, and of our knowledge that functional diseases are sometimes found to be of a graver nature than was at first thought to be the case, and that they are really dependent upon organic or structural changes in the parts concerned. You will readily understand that an organ which is in an early stage of permanent disease may show marked changes in its workings long before we can detect, by the still imperfect methods which are at our command, any gross structural alteration in it. And sometimes the very reverse of this will obtain, organic disease of a very serious nature existing for a long time and giving rise to very few symptoms, or to such slight troubles that no notice has been taken of them. As an illustration of the first fact one may cite a cancer of the stomach. Here, for a considerable time before the real mischief reveals itself by unequivocal signs, grave disturbances of digestion may occur, and the physician, however skilled he may happen to be, may find himself at a loss to account for the persistency of symptoms which prevail in spite of his treatment. Of the organic disease not revealing itself by any symptoms of striking import let me take cancer as affecting the lower part of one of the bowels—say, the colon. Here an insidious growth may be involving the gut, but, owing to the fact that it is situated at a point where nearly all the processes of digestion and of absorption of food have been completed, little disturbance results; all that need happen is that by a lessening of the calibre of the bowel at the spot of disease a gradual constipation may ensue. This constipation may be so slight that it may be unnoticed, until some day it becomes extreme, and quite suddenly symptoms of absolute obstruction of the bowels present themselves. Thus a dire organic disease may lie hidden, and its first disclosure may indicate a condition of the most extreme danger. I operated upon a man some years ago who was a typical example of what I am speaking of. He was elderly, very fat, and well nourished, and apparently was in the rudest health. After noticing that he had some difficulty in obtaining actions of the bowels, which he overcame by taking strong purgative medicines, he quickly developed symptoms of obstruction, and on operating I found him to be suffering from a ring of hard cancer which was occluding the bowel. This must have been present for a long time before it caused trouble, and serves as a good instance of the point I have been desirous of speaking about.

You will naturally ask me how we proceed in order to find out whether the disease we are investigating is of a functional or of an organic nature. The answer to this embraces the whole realm of diagnosis, one of the most difficult parts of the physician's knowledge. To be brief, it is done by a careful investigation and examination of the patient, in which all the knowledge which has been acquired of pathological anatomy and the use of instruments for this purpose is brought to bear by the examiner. There is a constant addition of these instruments being made, and the medical student of the period is being trained in their application. If anything, the tendency now is to lean too much upon these aids to diagnosis (or discovery of the nature of an illness), and to neglect the full use of the judgment, guided by observation of symptoms. If a man will be content to make a patient inquiry into all the sufferings of his patient, and will observe with care the indications of disease afforded by the face and skin, the sputum, the breathing, &c., and will then use such instruments and chemicals as may be called for, he will be as perfect a physician as can be found; if he relies on one-half of his means of discovery he will only fail in his results. A well-trained man seldom fails from want of knowledge; it is in the failure to use what he knows that he meets with want of success. The same thing applies to a nurse who has received proper instruction. If she is lazy, and will not take the trouble to do more than her duty calls for, she will fail in her vocation; if she goes beyond mere routine and lays herself out to do the innumerable acts of kindness and of sympathy which she has a chance of, she will be a signal success in life. She is not responsible for the opinion entertained by the medical attendant of the nature of the disease for which her services are required; her duties are confined to a loyal co-operation with him in seeing that his directions are carried out to the fullest extent, and in the performance of her work of mercy in the kindliest and most soothing manner. If she takes this view of her duties she is an invaluable help to both the doctor and the patient.

Now I want to say a few words about the subjects of heredity and temperaments. The person who takes upon himself to treat disease cannot be too careful to make inquiries on these points, for the life of any man or woman is dominated by the inheritance which has been bequeathed to him by his forbears. The difficulty here lies in obtaining any information which is of a trustworthy character, for few people are willing to allow that their relations have shown evidences of constitutional diseases, and many others know little or nothing of their kinsmen; and yet the greatest value attaches to information as to family health history, the fact being that tendency to many diseases is as certainly inherited as are peculiarities of character and of features. See how careful breeders of animals are in this respect! They know that by careful selection they can secure the perpetuation of a certain "breed" with all the best characteristics of the "strain" they are seeking to perpetuate. Men and women cannot be guarded against adverse influences in this way, so there is often the perpetuation in a family of dispositions to certain diseases just as there is of mental characteristics.

Both doctors and nurses certainly have much to do with the physical and mental characters of the individuals under their charge, and could often wish that they had some power of modifying or of changing them. Thus, for instance, that temperament which is called the nervous one is a disposition which gives us an infinite amount of trouble to deal with. The term does not necessarily imply timidity. People who possess this temperament are often very courageous, but they are quick and anxious in disposition and are excitable, and they make restless and difficult patients. They are intelligent persons, and suffer more than the dull and phlegmatic ones in illness, for there is no doubt but that the highly-strung, intelligent being pays a penalty in suffering for the greater acuteness of his intellect, and for the keener appreciation he has of the real nature of things than is possessed by his less thoughtful neighbours.

The two other notable types to be met with are the sanguine and the lymphatic ones. These are the very opposites of one another, the sanguine person being bright, quick in action, hopeful, and impulsive; while the lymphatic one is dull, slow, and lethargic. Strictly speaking, dispositions ought to be shown in the formation of bodies, the colour of the skin, and in the actions of the mind; you will find that to a great extent this does obtain. The person who is quick to recognise the dispositions of others, and who acts on this knowledge, becomes very successful in life, especially if his calling brings him into contact with his fellow-man. Inasmuch as the nurse has to perform many duties which require tact and judgment you will find it a useful accomplishment to acquire some insight into these matters of dispositions and of temperaments.

### The Sick and Wounded in the "Winifredian."

CHAT WITH THE SISTER IN CHARGE.

On a sunshiny March morning, with a feeling of spring in the air, and birds (mostly sparrows !) twittering in the tops of the budding chestnuts, I went down to Woolwich to see and hear something of the returned soldiers—the sick and wounded who had just arrived in the " Winifredian." They had arrived at Southampton before their time (thus losing the chance provided by the Absent-minded Beggar Fund of sending free telegrams to their friends) and had been brought to Well Hall Station, whence they were conveyed to the Herbert Hospital.

It was a pleasant walk up the hill, past long lines of soldiers, red and black, with here and there a touch of khaki, for my visit was happily timed, and the troops had turned out for a preliminary parade.

"The Queen does not come till Thursday, does she?" I asked an ancient by the roadside; "why are they all out to-day?"

"They're just putting them through it, like," he answered, cheerily; and at the hospital, too, great preparations were afoot. What with the Queen's visit, and the constant changes in the staff owing to so many, both doctors and sisters, being ordered to the front, the place was not, as I naively suggested, "upset," but "getting ready to put everything in order," which sounds better, and is more dignified. Two sisters sail in the "Briton," and one of them kindly ran and put on her blue and red cloak for me to see.

"About one hundred and fifty men are here from the 'Winifredian'; a few, too serious to be moved so far, were left at Netley; the whole number was 170, of whom some were marines, who are sent to the naval hospital at Gosport. Those at the Herbert Hospital are suffering chiefly from gunshot wounds, dysentery, and rheumatism, and some are convalescent enteric fever patients.

What the Men Say About the Nursing on the "Winifredian."

"It couldn't have been better," they agreed, and I saw a good many scattered over the hospital in the different wards, who all said: "the sisters did everything they could for us, and we enjoyed ourselves thoroughly." "The voyage was really the best cure they could possibly have had," added the sister, who, in the little red military-looking cape, with what the street urchins of Woolwich call "a blooming big half-crown," was most kindly taking me round the wards; "it gave the men the rest and quiet they needed, and with good food, it is wonderful how they have recovered during the time."

"So that they are really almost convalescent by the time they arrive?"

"A good many are, but many still require a good deal of attention."

Almost more suffering seems to have been caused by exposure to damp than by wounds. "We were seven hours in the river," one man said, "and then sun-dried, and wet again at night, when it poured with rain. We couldn't lie down, but sat back to back, four of us, and kept one another warm." One poor fellow belonging to the Medical Corps, is quite crippled with rheumatism, his hands being, as he expressed it, "properly twisted"; but he managed to unfold a red pocket handkerchief to show me, with great pride, the legend "Absent-minded Beggar" in the middle.

"Do you want to go back to the front?" I asked.

"I'd as lief be there as here," said one; and another was quite apologetic because, he pleaded, "you couldn't feel like going back when you were sick." Many have gone on in the ranks, hardly able to stand, rather than report themselves sick and be sent home.

"You've had a rough time out there?"

"Yes, but we musn't grumble, and we've had so much done for us; in fact, he added, with a twinkle in his eye, "Tommy's spoilt. Kipling knows us," he went on, "he brought us out these," and he produced a box of cigarettes, inscribed, both box and "weed," with the immortal words, "We are absent-minded; it's each one for himself; we bury a hundred and sixty of our comrades, and then we go away and forget." But his serious face belied his words; he did not forget.

"I couldn't tell you the sights we saw," said another; "it was like mowing down so many bushes." No wonder the Princess said, "This terrible war; this horrible war!"

What the Sister in Charge Says.

After many inquiries I found the nurse in whose care the men made their voyage from the Cape. She remains for little while in London, and probably sails again with an outward-bound hospital ship. The arrangements on board were, she said, excellent; everything was most comfortable, and she could not speak highly enough of the kindness shown by all, from the captain down to the lowest rank.

"I was rather afraid," she continued, "that with such frequent calls on the steward's resources for beef tea, milk and eggs, and all the things one is continually wanting for the sick, the patience of that department might become exhausted; but everything was done most willingly and cheerfully throughout the voyage."

"How long did the voyage take?" I asked.

"Eighteen days. We sailed on Majuba Day, when the premature reports of the relief of Ladysmith were received, so we left amid great excitement, with a blare of trumpets."

"Had you any very serious cases on board?"

"Yes, a few. We had no deaths. But wounds heal wonderfully quickly in South Africa; and one young fellow of twenty, who had his leg amputated quite high up in the thigh before leaving the Cape, was able to get about quite nicely on board. The ship's carpenter made him a pair of crutches, which the Absent-minded Beggar Fund replaced by a lighter pair when we arrived at Southampton, and one of the Prince of Wales's walking-sticks was given him also?"

"Is it true that you can have a bullet through your arm and not feel it?"

"You feel a sharp pinch, and then it is numbed. One man has two bullets in his leg, which he feels when he walks; he feels them dropping, but he is fairly comfortable, they give no pain, and the doctors think the risk of removing them too great, at any rate for the present."

It was owing to the sister in charge proper, if one may so speak, being taken ill on the voyage, that Mrs. Stuart, who has been a nurse at East London, became responsible for the hundred and seventy patients, and such good care did she take of them, that at Southampton they presented her with a dressing-case and an address "from a few of the Queen's soldiers and sailors, to Sister Stuart," in the hope that she would live long, and that the sufferings of many other soldiers and sailors might be alleviated by her as their's had been. This was a great pleasure and a great surprise to Mrs. Stuart, and the gratitude of the men touched her deeply.

Mrs. Stuart, like everyone else, including the men in the Herbert Hospital, was looking forward to the Queen's visit to Woolwich; and the words of the Irish soldier whom I saw lying in bed, watching his comrades polishing the hospital windows, may fitly conclude this account: "Begorrah! we're going to be up-to-date for the Queen!"

### The Title of Sister from a Nurse's Point of View.

By a Correspondent.

The Bristol Board of Guardians have recently taken exception to the title of Sister. I am sorry to say that they are not alone in their opinions. The question came up at the board meeting of a larger union a very short time ago. For the nonce that union shall be nameless. There had been so few applicants for the post of "charge nurse" that the matron ventured to suggest that they should style them "Sister." The guardians shook their heads, and after some discussion about the value and the meaning of the title, one Guardian said: "Oh, give them another £5 a year; that will bring more applicants," and so the matter dropped.

### Why not Nursing Sister?

Now, as they have given us their view of the question, would it be amiss for us to give them ours? I cannot think that they have looked at it from every point of view. Why should the title always be associated with purely religious bodies? With how many professions is the title of doctor associated? We have Doctor of Music, Doctor of Divinity, Doctor of Medicine, &c. What does the title imply? Simply that the member holding it has passed a certain examination, attained a certain degree of efficiency in that particular art. Then why should we not have nursing sisters as well as religious sisters?

There are the Army Nursing Sisters; they are not a religious body, only in the same sense as I trust all nurses are. Surely the story of Florence Nightingale and her arduous training is too well known for anyone to suppose "that a desire to do good," a religious bent of mind, are the only qualifications needed to insure the lives of our soldiers being placed in their hands.

### The Need of the Title.

Do away with the title of sister and we have nothing to indicate at what stage of our career we have arrived between that of a probationer, nurse, and a matron. Some such title is needed. All we ask for is one which will convey in a word our professional rank. The youngest recruit is addressed in the wards as nurse. It would sound very unpleasant to hear ourselves called "head nurse." Sister is a most appropriate term. In the mind of the medical profession the meaning and the use of the word is clear, and what we want to establish in the minds of the general public, and of all those who have either the power to confer or refuse it, is that the title of Sister means that any member of the nursing profession holding it has gone through a given training, successfully passed an examination, obtained a certificate, and is filling some position of responsibility, such as charge nurse in a training school, or in another equally important sphere. Though there is great diversity in the training of the nurses filling such offices and the qualifications held by them, one certificate for general training is required by all, together with special fitness and aptitude for the control and teaching of others.

### Our Right.

Also, though the religious bodies may have the prior claim, still the title has been too long associated with our great voluntary hospitals, and many workhouse infirmary training schools, for anyone to contend that nurses have no right to it, or that it makes them appear something different from what they really are. It is not the only word in the English language which has more than one meaning and usage.

### The Phrase "Charge Nurse."

Returning to the phrase "charge nurse," a little inquiry into the matter reveals the fact that in different hospitals "charge nurse" and "staff nurse" imply much the same position, viz., that of one who has only a limited sphere, and works directly under the supervision of another head. In small unions many nurses working under the superintendent nurse are often called charge nurse or assistant nurse, but their position is not equivalent to that of a sister in a training school. A sister has no other heads than the doctors and matron, then why not give some title of recognition of her ability and status? To my mind this is one of the questions which is bound to be settled sooner or later to our satisfaction if we are not too apathetic about it. Only let us do all in our power as occasion arises to further our end by trying to convince those in authority, or any lay people, of the fallacy of their theory. Those nurses who are not closely concerned, if they are convinced of the justice of the request, should not withhold their help.

### Novelties for Nurses.

### A USEFUL INVENTION.

We have received a sample of a new sanitary Bandelet, which is an ingenious and original invention, and deserving at least a trial. The Bandelet is composed of linen folded and containing a powdery substance said to be absorbent. They are shaped on sound principles, and will no doubt become popular when their merits become more known. It is patented by Mr. Louis Nelicker, 29, Bedford Road, Rock-ferry, Cheshire.

### "PAROCHIAL" MARMALADE.

Since the publication of "Oliver Twist," the word "parochial," even when not disguised by Mr. Bumble's familiar modification of it, has seldom been employed in any sense synonymous with excellence, and we are, therefore, the more glad to point out that this may now be done. Those of our readers who are able to appreciate the qualities of really genuine marmalade, made of nothing but oranges, lemons, and cane sugar, and absolutely free from any of the admixtures of carrot, gelatine, or glucose, which, together with matters still more objectionable, are commonly believed to hold an important place in some manufactories, will thank us for calling their attention to an enterprise which for the last few years has been successfully carried out by Miss Watson, of Woodside Parsonage, King's Langley, R.S.O., Herts. This lady, originally moved by a guest's admiration of her home-made marmalade, was induced to make some for sale among friends, with the twofold purpose of establishing a village industry which should afford employment under her direction for some of her father's parishioners, and of devoting any profits which might accrue to the support of the parochial charities, the cottage hospital, the coal club, the clothing club, and so forth. The sale, which commenced with 117 lb. in 1893, reached 11,048 lb. in 1899, and left a profit of £82 14s. 3d. Miss Watson now gives her customers a voice in the distribution of this profit, allowing purchasers of fifty pounds of marmalade to indicate any charity to which they would like five shillings to be given, and so on in proportion. Lately a summer manufacture of jam has been added to that of the marmalade, and for both the same standard of purity and excellence is maintained. Everything is done under the strictest personal supervision of Miss Watson herself, in a cottage hired for the purpose, and by residents in the village; and, were it not that the name "parochial" seems now to be established, we should be disposed to suggest that "home made" might be substituted for it with advantage. However this may be, there can be no doubt that those who support Miss Watson's undertaking will find themselves put in possession of a marmalade of a quality such as, as far as we know, is not to be bought elsewhere.

### Royal National Pension Fund for Nurses.

### THIRTEENTH ANNUAL MEETING.

The thirteenth annual general meeting of the members of the Royal National Pension Fund for Nurses was held at River Plate House, Finsbury Circus, E.C., on Thursday, March 15th, the founder of the Fund, Sir Henry Burdett, K.C.B., deputy-chairman, presiding in the absence of Mr. Everard A. Hambro, the chairman. Supporting the chairman were the following members of the Council: Sir W. Broadbent, Bart., M.D., F.R.S., F.R.C.P., Mr. Thomas Bryant, F.R.C.S., Mr. Walter S. M. Burns, Mr. C. Eric Hambro, Dr. Herbert P. Hawkins, F.R.C.P., Mr. J. Pierpont Morgan, jun., Mr. G. Norman, Mr. E. C. Perry, F.R.C.P., Mr. Edward Rawlings, and Mr. John Watney. Amongst others present were: Mr. Alfred J. Waley, Mr. George King, F.I.A., F.S.A., Mr. Perceval A. Nairne, chairman of the Seamen's Hospital, Greenwich; Miss R. Pritchard, hon. secretary of the Benevolent Fund; Mrs. Farmer, the secretary of that Fund; Miss Monk, matron of King's College Hospital; Miss Mabel Cave, matron of Westminster Hospital; Miss Young, matron of Guy's Hospital; Miss Shirley, lady superintendent of the North Staffordshire Nursing Institution; Miss Evans, of Guy's Hospital; Mr. H. M. Chester, treasurer of the Royal Surrey Hospital; and Mr. P. Michelli, secretary of the Seamen's Hospital.

Sir Henry Burdett, in moving the adoption of the report and balance-sheet, said: Ladies and gentlemen, it is now my privilege to propose the adoption of the annual report. I think it is a gratifying fact that the investments of this Fund have reached a sum as large as to exceed half a million sterling at a time when old age pensions have been so much before the public. We have heard and read a good deal about the provision of old age pensions for all classes of the community, but in my view, and I think this Fund is a sound reason for the faith that is in me, it would be a calamity to have any system of old age pensions which had not a thrift basis. I do not think myself, and I have studied this question for more than thirty years, and can claim an intimate knowledge of the feelings and the powers of saving of the humbler class of citizens, that any proposal to give old-age pensions to everybody who had attained a certain age, however much it might be fenced around with conditions to protect it from abuse, would be anything but an evil to the nation. I say this because I believe that members of Parliament and politicians generally who have approached this question, and who necessarily regard it from the point of view of what appears to be popular, have failed to realise the effect of universal pensions upon the character of the people of the United Kingdom. I think, too, they fail altogether to realise the effects of the Education Acts which were passed by Mr. Forster some thirty years ago, and the circumstances of the people during those thirty years. The effect of those Education Acts has been material upon the well-being, the character, the views, and intelligence of the humbler classes of citizens throughout this realm. You may depend upon it (and I claim that I have proved it on a previous occasion) that it is possible to so arrange the basis of old-age pensions that the humblest wage-earner—the man who may not get more than 18s. a week—may give a small sum weekly out of his earnings. This would have a humanising and beneficial influence upon him as an individual, and also be helpful from the point of view of statesmanship, for it would make him more of a man, it would make him more self-respecting, and therefore more useful and more honourable as a citizen of this great Empire. I do not think it is at all out of place to make remarks of this kind at a meeting like that we have to-day, when we celebrate the glorious result of ten years' work, as represented by 500,000 sovereigns collected to secure pensions on a thrift basis. These 500,000 sovereigns have been invested by one class of women workers only. It has been said that nurses are above the average intelligence of women workers, and that their earnings are above the average; but that is not substantially accurate. They are drawn from all classes, and their earnings, taken on an average, are not very high; and another fact should be remembered. The nurses serve, for the most part, in large institutions, they are resident in great buildings, and there is a tendency among their friends to believe that they are well paid, and to cause those friends to continually encroach upon the limited earnings of the nurses and expect them to help to support the family, which in the old days, was always regarded as the duty of the male portion of the community. Remembering all that, I think you will agree with me that there is very little in any criticism of that kind. I know this, that if nurses, as they have proved by the progress of this Fund, can afford and will cheerfully contribute out of their earnings towards a fund which will provide for them in the days of sickness and for the time when their working days are over, there is no class, male or female, in this country that cannot do its share, and would not, on the whole, gladly contribute to any pension scheme which may one day be established. I am definitely of opinion that we ought to have a pension fund, and that everybody should be helped to save, so that they could maintain their independence after their working days were over. We are justly proud of our country, but how much prouder of it might we be if we could be assured that the humblest of its citizens, after their honourable lives of toil, would have a certainty of being able to maintain themselves reasonably well in comfort and in happiness independent of institutional control and outside the workhouse, enjoying the good air and the sunlight with the best in the land. That is an ideal which I hope will be put into practice, but I would much sooner have another half century go by without any scheme of old-age pensions being put into force than by hasty legislation have the well-being of the whole population imperilled by some system of pensions which gave to all alike, and which entailed an enormous tax upon every section of the community, particularly that section maintaining itself by labour. Now I have to go a little into detail with regard to the Royal National Pension Fund for Nurses. I find we have had during 1899 a larger number of policies issued than in almost any year in our history, they having amounted to no less than 811, and the premium income money actually contributed by nurses during the last twelve months is no less than £70,000. The invested funds, as I have told you, amount to, in round numbers, £523,000. The rate which has been earned upon the actual new investments is no less than £4 9s. 6d., the average rate on the whole sum invested is £4 1s. 7d., and the average rate for the past five years has been £4 4s. 2d. per cent. Here I would like to emphasise and, on behalf of the Council and of the nurses and of those interested in their welfare, to express our sense of deep obligation to the eminent financiers and City gentlemen who are so untiring in all the trouble they take to see that the maximum sum is earned and the soundest security obtained for the nurses' savings.

During the year we paid away £1,297 in sick pay, and the ladies who are present to-day representing the large hospitals know what an immense boon sick pay represents to working nurses; and I am glad to say the danger that threatened this Fund a few years ago has passed away through the care and skill and devotion of our actuary and of the eminent medical gentlemen on the Council. They have succeeded in putting a stop to an almost natural idea that whenever nurses got a little tired they were quite justified in taking a holiday, and drawing from the sick fund the necessary amount to defray expenses. That is not the object of sick pay. Sick pay is a system by which every working nurse who enters for it is entitled, when incapacitated by illness, to receive a weekly allowance, so that she may be provided for in comfort and go away and have needful change and rest. To apply that, with somewhat loose morality, to a system whereby everybody who felt a little tired and wanted a holiday was able to draw the necessary expenses, would be to make any attempt to provide sick pay an impossibility. Actuaries have to work from actual figures. Those figures are derived from the actual illnesses which affect certain classes of the population; and, after all, the provision of sick pay is as much a business matter as the provision of interest on stocks.

We have paid away in pensions last year, although we have practically only been working eleven years, over £3,600. We have, further, with the aid of those gentlemen I have already referred to, made certain changes in our investments which have enabled us to put by on the investment reserve fund a sum which amounts to £7,279. Finally, as you know, there are no expenses connected with this Fund for management in the ordinary sense of the word. The Council is entirely honorary, and we have a great deal of assistance given us by medical men and others, so that our working expenses only amount to 3·74 per cent. That is a very small sum, much smaller than is usually the case in businesses of this description, and I am quite sure you will agree with me that it reflects the greatest credit upon our officers and office staff, and especially upon Mr. Dick, the secretary, who is untiring in his devotion to this Fund, and who has the absolute confidence of the Council. I think he deserves and largely possesses the affection of the nurses, for he has given up the best years of his life and is devoting all his energies to making the Fund not only a success but of real practical use and helpfulness to the nurses and to the institutions that employ them.

We have all a great interest in the war, and it is constantly in our mind. I think it may be of interest to you to know that a great number of our policy-holders are serving in South Africa, for at the present time more than seventy are nursing the sick and wounded at the seat of the war. We have also the pleasure of stating, and I personally have great pleasure in recognising the fact, that a member of our Council (the Hon. Egremont Mills) is at the front taking his part as an officer in the fighting line. That just shows incidentally how widespread is the interest in this war, and how all classes have participated in putting their shoulder to the wheel to bring the war to a speedy termination, and to uphold the flag throughout South Africa.

We have made a new advance in reference to federation, and many institutions have entered into the scheme by which they pay a certain proportion of the premiums, the nurses paying the other proportion. You will remember that when this Fund was first started it was stated that throughout the country there was an understanding that every nurse who remained sufficiently long in an institution should get a pension. In practice, however, that worked out that no nurse ever got a pension. Some left before the pension could be claimed. It is not very difficult to see how this came about, especially to those of us who have spent many years in the management of hospitals. A nurse enters a hospital and does devoted work, and whilst she might remain, after a certain number of years the council changed, and those who engaged the employés originally had no means of recalling all the good services rendered, and when the time for retirement came the result used to be most unjust and inadequate owing to the general feeling which pervaded committees that they were the trustees of public funds, and really could not make an annual charge by way of pension. They gave a donation of £20 or £30 to end the matter. This Fund by instituting the federation scheme (which embodies the thrift basis on which it is built up), whereby the nurse pays a proportion of the premium, the institution paying the other, is remarkably beneficial both to institutions and to nurses. Any institution which federates to the Royal National Pension Fund knows that so far as the question of sick pay allowance is concerned it is absolutely secure. The charge upon the institution for the whole of its staff is a small amount. It is paid annually in the ordinary course, and everybody is made happy and secure. I, therefore, consider that the federation plan is one well worthy the consideration of the managers of every institution for the relief of the sick throughout the country. During the year we have had several institutions federated, and the movement is extending, and I hope will ultimately include every recognised institution. I have only one other thing to say.

During last year the Princess of Wales received the nurses at Marlborough House, and the interest which was taken by their Royal Highnesses in the nurses on that occasion and which they have habitually shown in this Fund has been one of the greatest aids to its success. They have the secretary's report every year. It is a most interesting document, but somewhat complicated and full of figures. Last year it was read by their Royal Highnesses right through, and they were immensely struck and much interested in its contents, as was shown by the questions they asked after they had perused the document. They were surprised at the extraordinary results which the report revealed, and that throughout the Fund, in every department, there was a progressive increase, exhibiting a wholesome and healthy life.

It is a remarkable fact that this year, though it is contrary to all actual calculations, we have had a smaller number of withdrawals than usual, much smaller, in fact, than in any former year. Some people regard withdrawals as a testimony to the failure of the Fund from the point of view that they show people cannot keep up the premiums, or have not sufficient grit to go on saving. But the proportion of withdrawals to the number of new policies issued is a relatively small one, and you must remember that this plan of withdrawals which we have permitted enables the nurse who joins the Fund to treat it absolutely as a savings bank, because she gets compound interest on her money when she takes it out. The system has made many nurses surprisingly wealthy, for they would not otherwise appreciate how a small sum grows and grows into a considerable one; and when I tell you that £16,000 has been paid back to the nurses during the year, you will recognise it fulfils a very useful purpose. Of the causes of withdrawal there are several. The largest cause is marriage, upwards of 20 per cent. of the nurses getting married, and when they go away they take a nice sum as a little dowry, or to get a handsome trousseau. Some, I think, withdraw because of changes in the circumstances of their families. Difficulties may beset a parent or sister or brother, and there comes a claim upon the nurse. She naturally feels interested in her friends; still, I wish, on the whole, we could impress upon the nurses the fact that the money they put into a Fund like this is money in trust to obtain for them when they are past work or incapacitated the security that they will not burden their friends, but maintain themselves in reasonable comfort. Therefore, the money put into this Fund by the nurses ought not to be utilised for the purpose of paying some liability incurred, perhaps by a male member of the family. Such savings should not be taken and the nurse left without anything to fall back upon when she becomes helpless.

I rejoice to think that we have so speedily reached the time when we have more than half a million soundly invested, and everybody who is interested in the treatment received at our hospitals and in the welfare of those who serve and those who suffer much thank God that such a Fund has been established and is now placed upon so sound a foundation, and that it must continue to extend throughout the British Empire, and the Royal National Pension Fund must continue to have an increased amount of money at its disposal, and so extend its power for good by securing the happiness of a class of people with whom everybody who is healthy and well must have sympathy. I have great pleasure in moving the adoption of the report, and in calling upon Sir William Broadbent, who has given up his consulting practice to-day in order to be with us.

Sir William Broadbent, Bart., M.D., F.R.S., F.R.C.P., in seconding the motion for the adoption of the report, said: It must be clear to everyone that it is a subject for congratulation that the invested funds now amount to the large sum of half a million, giving security for the payment of the annuities which the Fund guarantees. Up to the present a comparatively small number of claims have arisen. Those claims will increase in number and the demands upon the funds will increase in amount, and it is most satisfactory to see that all these future claims are so amply provided for by the sum which has been accumulated. I can speak more freely on the subject of financial matters as they are dealt with by other members of the Council, and one cannot but admire the business capacity—one could almost say the genius—which has brought the funds to their present level, and which has secured the surprising rate of interest which you have heard from Sir Henry Burdett.

A very important point in this arrangement is that it becomes not merely provision for old age, but, as Sir Henry has well put it, it secures to nurses a return of their money actually with compound interest at any time. There is no risk. It is not as if when the power to pay a premium ceases, or from any cause whatever the necessity for the pension ceases, the money paid in was lost. It goes back to them, and with better interest than the nurses themselves can command. The sick fund is an extremely important part of the institution. Nurses are, almost more than any other class, liable to contract severe illness in the discharge of their duties, and to be able to secure something like an adequate provision for the period when they are incapacitated for work and be away for their health is very satisfactory. This is an extremely important part of the work, and I am glad to hear that it has been safeguarded in the way described, and that it is not looked upon as a mere holiday fund. That is a danger which one foresaw from the first. Many nurses have proved the value of this part of the Fund, and I believe I am stating correctly that not only does this part of the work benefit nurses directly, but it helps them indirectly. Many ladies, like Lady Rothschild, take a great interest in nurses who are disabled in the discharge of their duties, and the interest of persons in their position is one of those indirect and incalculable benefits which this Fund confers. I can only say that it has given me very great pleasure to come here and take part in this meeting, and to congratulate ourselves and the nurses and Sir Henry Burdett on the very remarkable success which has attended the Fund.

The resolution adopting the report and balance-sheet was submitted and carried unanimously.

Mr. P. A. Nairne proposed the re-election of the retiring members of the Council—Mr. A. C. de Rothschild, Mr. John Watney, Mr. Thomas Bryant, F.R.C.S., Mr. G. Norman, and Dr. E. C. Perry, F.R.C.P. In doing this, he said: It has been of the greatest possible advantage to this institution that ever since its commencement the seats on the Council have been filled by men of business and of the highest standing in the City of London, as well as by medical gentlemen of the greatest eminence. The work, although often dealing with small amounts, is of an intricate character. The work of councils and committees of hospitals has recently been very much eased in the matter of providing pensions for their old and deserving nurses who have done many years of continuous service in the one institution. Councils and committees can now deal with this question through this society with very much less difficulty than hitherto. I feel that the nurses who are assured by this Fund owe a great measure of thanks to the Council of this institution for the manner in which it is carried on and the benefits it is extending to all its members.

Mr. A. J. Waley formally seconded the resolution, which was carried *nem. con.*

On the motion of Mr. G. Norman, seconded by Mr. Pierpont Morgan, Mr. Frederick Whinney, F.C.A., was unanimously re-elected auditor, the Chairman referring to the satisfaction expressed by that gentleman at the way in which the accounts were kept.

Mr. Thomas Bryant, F.R.C.S., proposed a vote of thanks to the medical referee. He said: It gives me very much pleasure to propose a vote of thanks to the medical referee, Dr. Potter, for his report on the sick assurance fund for 1899, and from what we have heard we know he has done exceptionally good work not only in carefully guarding the Fund but also in placing it on its satisfactory basis. One does feel that our medical referee meets everyone in a fair and competent spirit, and that he has been of very great value to this Fund. I have great pleasure in proposing this vote of thanks.

Dr. H. P. Hawkins, F.R.C.P., seconded the motion, which was unanimously adopted.

Sir Henry Burdett: I should like to say a few words about the Junius S. Morgan Benevolent Fund. Not only are nurses provided for in the days of sickness, but by this benevolent fund, in memory of Mr. Junius S. Morgan, who gave the first sum of money to enable premiums to be kept up when nurses were in difficulties, it enables a committee of ladies, of which Lady Rothschild is president, to take all the cases of our members who become in any temporary distress or who may become prematurely disabled, and afford assistance where needed. In one case, a nurse—quite a young woman—became temporarily quite disabled. They took care of her, looked after her, and now after some year and a half she had been restored to health. Her premiums have been kept up, and she is perfectly happy and safe. And where there are older members who have pensions which are not quite enough the ladies look after them. There were some wonderful instances of the interest taken in the work by ladies, and we always have some to come forward and volunteer assistance. No one could attend the meetings of the committee without being impressed with the marvellous amount of good work which was accomplished with a relatively small fund.

The Scrutineers (Mr. C. Eric Hambro and Mr. Walter Burns) announced that the voting for representatives of the policy-holders on the Council had resulted in the re-election of the retiring members as follows: Miss Mabel Cave, 1,237 votes; Miss E. Fisher, 1,244 votes; Miss L. M. Gordon, 1,256 votes; Miss K. H. Monk, 1,241 votes; Miss M. Shirley, 1,233 votes; Miss E. Vincent, 1,239 votes; Miss E. Young, 1,242 votes; 4,453 voting cards had been sent out and 26 were spoiled.

Mr. George King, F.I.A.: Very few words from me will suffice to commend to you the motion which I have to propose, viz., a vote of thanks to Sir Henry Burdett for his conduct in the chair. He has explained to you fully and lucidly the progress of the Fund, and my mind goes back to the early days when Sir Henry had hard work and great anxiety in connection with it, and only by his resolution and steadfastness of purpose was success achieved. We must not forget the work of the past. The chief feature of the Fund at present is the large accumulations of money. We hear that now more than half a million is invested, and invested at a good rate of interest. That is exceedingly satisfactory, but it is only a means to an end. There is something far better than the mere accumulation of money and that is its useful disbursement, and I am very pleased to see the increase that is made in the amount of annuities paid out. It is not every institution that is glad to see an increase in the annuities paid, but the Royal National Pension Fund for Nurses is so organised that the whole of the expenditure is provided for, and we can look forward with perfect equanimity to a very large increase in this account. During the past year it was 30 per cent., and it will go on rapidly until it reaches the premium income, but it is entirely provided for in the mortality tables, and the larger it is the more satisfactory it will be. Some people of morbid minds have spent their efforts in building mausoleums for themselves. Sir Henry Burdett is preparing his own monument in this Fund. Long may he be connected with it and give it the benefit of his labours, his energy, and skill.

Mr. WALTER S. M. BURNS seconded. We all know, he said, the absolutely vital part which Sir Henry has taken in forming and carrying on the Fund. The great success and great popularity of the Fund was due, in no small measure to his efforts and the time he had spent on this noble work. He had great pleasure in seconding the vote of thanks to him, not only for presiding on this occasion but also for the help rendered on other occasions as well.

The vote having been cordially agreed to,

Sir HENRY BURDETT replied: I thank you very much. I feel, of course, great pleasure, I may say joy, in the success of this Fund, because, having presided for something like fifteen years in various hospitals, and had charge of the staff, I have had opportunities of realising the enormous amount of good which the Fund was capable of doing. So far as any thanks are due to me to-day, those thanks are more deserved by my friend Mr. Hambro and the other members of the Council and the officers of this Fund. I think if the truth were really to be expressed of the work done by the Council, I do probably less than the younger members, the sons of the gentlemen who founded this Fund. It is always a pleasure to meet them, because every time I go to the Council, and see them working so cheerfully, I realise what an enormous interest they have in it. Last year some question of payment for assistance in looking after the Benevolent Fund came up, and two of the sons of the founders met that expense. That expresses more than any words of mine how genuinely interested they are in it. They believe in it, and feel that it will endure. All honour to them, rather than to me, because they are bearing the heat and the burden of the day. I may with propriety ask you to thank the secretary (Mr. Dick), who last year had a great deal of extra work owing to the reception at Marlborough House. He took a pride in showing that the organisation of the Pension Fund was complete, and although there were only nine days for the preparations, he courageously set to work to arrange for the reception of 1,200 nurses at Marlborough House. That was carried through successfully. All I can say is that I have been at every one of those receptions and we never had a more successful demonstration or one better organised, and the amount of work done, which meant not only ordinary office hours, is good testimony to the interest which Mr. Dick and his staff take in this Fund. The interest he takes deserves a most cordial vote of thanks, not only on your behalf, but on behalf of the nurses with whom he has to deal, and whose friend he is, as none know better than the nurses themselves.

The vote having been unanimously carried, and Mr L. H. M. DICK having returned thanks, the proceedings then terminated.

### The Invalid Children's Aid Association.

OWING to the sudden illness of the Marquis of Lorne, the chair at the annual meeting last week of the Invalid Children's Aid Association at Grosvenor House was taken by Mr. T. HOLMES, chairman of the committee.

The CHAIRMAN, in moving the adoption of the report, explained the objects of the association, which were aptly illustrated by an incident related by Mrs. GARRETT ANDERSON, M.D., in supporting the resolution. She was spending a holiday in a country village when one boy, in a quarrel, threw a glass bottle at another and cut the femoral artery. The injury was so severe that the following day the leg had to be amputated in order to save the lad's life. He was a bright, intelligent, well-grown little fellow of thirteen, and the accident prevented his earning a livelihood in the usual manner; but a lady visiting him discovered he had a talent for drawing, and he was taught wood carving. He is now a successful workman, and has been for years in steady employment. He is on the point of marriage, and thanks to the timely aid is a useful and happy member of the community. The hope and object of the society, Mrs. Garrett Anderson urged, was the individual care and training of the children isolated by accident or illness and to educate them into good and useful men and women. In conclusion, she emphasised the fact that the society does not interfere with or supplant parental obligations, but in a moment of overwhelming calamity it lends that helping hand which enables the family to meet and support the trial.

The Rev. M. R. NELLIGAN having begged for more workers,

The second resolution pledging the meeting to aid in the extension of the work and securing it more liberal financial support was proposed by Sir THOMAS SMITH, Bart. Sir Thomas spoke of the work of the association from personal experience. Many chronic cases, or cases requiring prolonged treatment, were, he said, of necessity put out of the general hospitals because there was no room for them. He had found such cases of his own wisely and kindly supervised and kindly attended by members of the society, with the result that in some cases complete recovery was attained.

Mrs. HUMPHRY WARD seconded the resolution, and availed herself of the opportunity afforded to call attention to the schools for invalid children—an offshoot of the I.C.A.A. now successfully established at the Passmore Edwards Settlement, Stepney.

Mrs. CRAIGIE pleaded for signatures in the "Golden Book" of the association. Friends signing their names in this volume promise help in exceptional cases, and may be applied to when such are found. Reports of the child's welfare and progress is regularly forwarded to her patron.

Mr. EDMUND OWEN, F.R.C.S., also spoke.

The election of the committee was proposed by Mr. LEVESON SCARTH, and Sir SAMUEL LEWIS seconded; and Sir CHARLES FREMANTLE, K.C.B., having proposed a vote of thanks to the chairman, it was resolved to send a telegram to the Duke of Westminster, who is now at the front, thanking him for the use of Grosvenor House for the occasion.

### To Nurses.

WE invite contributions from any of our readers, and shall be glad to pay for "Notes on News from the Nursing World," or for articles describing nursing experiences, or dealing with any nursing question from an original point of view. The minimum payment for contributions is 5s., but we welcome interesting contributions of a column, or a page, in length. It may be added that notices of entertainments, presentations, and deaths are not paid for, but, of course, we are always glad to receive them. All rejected manuscripts are returned in due course, and all payments for manuscripts used are made as early as possible at the beginning of each quarter.

### Nursing the Wounded at Naauwport.

By an Army Reserve Nursing Sister.

It is February 21st, and we are much nearer the front than we were this time last week. We arrived here a few days ago after thirty-five hours' travelling from Cape Town. The journey was a very hot and uninteresting one, and the night travelling was rather uncanny, because we felt that we were in an enemy's country. When we drew up at the stations horrid black men, whom I am sure were spies, kept peering into the windows to see if any officers or men were on board.

The country round Naauwport is not picturesque, but such lovely clouds and lights flit over the bare, weird-looking mountains that at times they make a beautiful background to our pretty camp. Lord Roberts was here last week, and we expect a visit again at any moment, as we are told he is coming to see the camp. I have been very busy making curtains for the cottage hospital, as we want everything to look very nice, and the stationmaster has sent me up some coloured prints to hang up, which will delight our sick "Tommies."

There are a great many bullock carts here taken from the Boers. Last night a train of ten passed, each drawn by sixteen oxen. It was such a pretty sight. The waggons are twice the size of English ones, and a third of the back of the back is covered like a carrier's cart, the fore part being left open.

### A Tropical Rainstorm.

On Saturday evening and night we had the worst rainstorm it has ever been my lot to see. We had a slight sandstorm more or less all day, and about six p.m. down came the rain, and for the first hour thunder and lightning accompanied it, the wind was very strong, and there were small lakes and rivers to be seen before we came off duty. When we went on duty in the morning we found several tents had been blown down by the wind in the night, and our patients were all scattered amongst other tents. The officers and orderlies had been up working all night that the poor patients should not suffer. At one p.m. over two hundred wounded came down from the Modder; they had all been wounded on Sunday and Monday week. They were more or less convalescent, but very tired, as they had travelled three days in bullock waggons and nearly twenty-four hours in the train. They are mostly Seaforths and Black Watch, but there are several from other regiments besides. Such fine fellows, and so brave.

### A Bed Once More.

It did one good to go and see them all so comfortable and happy in bed last night. Some had not seen a tent for two months, and the great majority of them had not been inside a bed for four months, but had been sleeping in the open. One fine-looking man, an Australian, said to me, "I hung on as long as I could. I wanted to finish the Boers, but my horse was shot under me and rolled on the top of me. I want to get back soon; we shall never be happy till we have settled the lot." We expect to be very busy shortly, as 100 more cases are now coming in, and marquees are being put up for 600 beds. We are to have a staff of twenty sisters. Some of the cases of enteric and dysentery which we are nursing now are rather bad.

### A NEW PRIVATE HOSPITAL IN MELBOURNE.

Miss Glover, formerly nurse at the Royal Hants County Hospital, Winchester, and at Queen Charlotte's Lying-in Hospital, London, has just opened a private hospital in East Melbourne. Miss Glover was for two years assistant matron of the Alfred Hospital, Melbourne, and for seven years lady superintendent of the Melbourne Trained Nurses' Homes.

### Appointments.

Gore Farm Hospital, Darenth.—Miss Jessie R. Warr has been appointed Assistant Matron. She was trained at the Evelina Hospital for Children for two years, and for three years at King's College Hospital. She has since been ward sister at Birmingham Infirmary, charge nurse at the South-Western Hospital, Stockwell; midwifery pupil at Kensington Infirmary; ward sister at Monsall Hospital, Manchester; night superintendent at Belfast Royal Hospital; and night sister at St. Mark's Hospital, City Road, London.

Chelsea House.—Miss Eleanor Barton has been appointed Matron of Chelsea House, the new convalescent home in connection with the Chelsea Infirmary at Westgate-on-Sea. Miss Barton was trained at St. Bartholomew's Hospital for two years, and at the Royal Hants County Hospital, and has for the last three years held the post of superintendent of the Nurses' Home at Chelsea Infirmary.

Poplar and Stepney Sick Asylum.—Miss Bessie Buller has been appointed Senior Assistant Matron. She was trained at the Bristol General Hospital, after which she held the post of sister of the casualty and out-patient department, and later that of sister of the female medical wards. She has since been night superintendent at the Swansea General and Eye Hospital.

Female Garrison Hospital, Portsmouth.—Miss Gertrude Weston has been appointed Matron. She is a "Nightingale" nurse, and was trained at St. Thomas's Hospital for four years, and afterwards at the British Lying-in Hospital, Endell Street. Miss Weston holds the L.O.S. certificate.

Altrincham Hospital, Cheshire.—Miss Fannie Smithies has been appointed Lady Superintendent. She was trained at Bolton Infirmary, and has since been matron at Bridgend Cottage Hospital and assistant lady superintendent at the Hull Royal Infirmary.

New Joint Infirmary Hospital, Elgin.—Miss F. M. Walker has been appointed Matron. She was trained at Ayr County Hospital, and had charge of it for three years. She has since been sister at Monsall Fever Hospital, Manchester.

Cottage Hospital, Twickenham.—Miss Quick has been appointed Matron. She was trained at the General Hospital, Nottingham. She was for some years at the National Hospital for Paralysis, Queen Square, Bloomsbury. Since leaving Nottingham she has been engaged in private nursing.

### Death in Our Ranks.

Great regret has been caused in Chester by the death of Miss Mary Frances Barrow, lady superintendent and matron of the Chester General Infirmary. Miss Barrow was appointed matron of the infirmary in 1881, when she succeeded Miss Osmond. Her career at the infirmary was marked by the great improvement she wrought not only in the nursing staff, but in the wards, eleven of which, owing to her exertions, were renovated and decorated. She founded the private nursing branch of the institution, which has been largely appreciated throughout the city. Owing to her efforts this particular branch is now one of the most important features of the institution, and proves a great source of revenue to its funds. No fewer than twelve or thirteen nurses are now employed in private nursing, and one of Miss Barrow's latest acts was the framing of a scheme for the re-adjustment of the nursing tariff and the higher remuneration of the outside nursing staff, whereby the department was brought into line with the best nursing institutions in the country. At the funeral, in addition to the Bishop of Chester, a large number of medical men and other well-known persons in the city, thirty members of the nursing staff were present, several of them contributing beautiful wreaths. Carriages were lent by several ladies for their use to and from the cemetery.

### Echoes from the Outside World.

AN OPEN LETTER TO A HOSPTIAL NURSE.

The British flag has been flying over Bloemfontein for a week, Lord Kitchener has occupied Prieska without opposition. On all sides the Free Staters are laying down their arms, appearing right glad to do so. Lord Roberts has issued a proclamation that any of the rebels who give up their guns and sign a declaration that they will not fight against the Queen again can return to their farms; but ex-President Steyn has endeavoured to stop this wholesale surrender by sending despatch riders warning the burghers that any who sign will be treated as traitors and be shot. President Kruger is said to have assured a deputation of Boers that he is going to continue to fight, and that he is also going to win. On the other side, Lord Wolseley—whose prophecy that we should enter the Free State capital before March 15th was true to the day—thinks that our troops will be within the walls of the Transvaal capital on May 15th. When we rule at Pretoria even Oom Paul will have to admit that he is not exactly winning. The two great anxieties left us are, chiefly, of course, Mafeking, which we had hoped ere now would have been relieved, and Johannesburg. According to the statement of Mr. Montague White, the Boer Consul in New York, the Boers are determined that we shall not possess the Gold Reef City, because it would make too good a base for our operations, and also it contains too much wealth. If the threat is fulfilled it would mean a loss of over a hundred million pounds. The Imperial Government, however, are issuing a document setting forth that any wanton destruction of the property of British subjects will be at the peril of the persons and the property of those by whom such destruction is committed, and this will tend to prevent the fulfilment of a threat which was probably not seriously meant.

---

A private letter from Chieveley from a young fellow who belongs to one of the Irregular Cavalry regiments shows the difficulties under which writing is done. He begins: "Excuse the paper being in such a dirty state, as I can only write from time to time, and when we are called away for duty I have to jam the paper into my pocket, which contains a pipe, tobacco, a piece of soap, an oil rag for cleaning my gun, &c., so that it does not get much chance of keeping clean." He was amongst those who had to retire after the last ineffectual attempt to get to Ladysmith. He says the fighting had been terrible. He had seen a coolie with both legs blown off, yet feeling the pain so little that he tried to crawl about to get to his master, and two or three horses disembowelled, which he describes as a most horrible sight. He says: "After leaving the battlefield we went out of sight behind a hill and grazed our cattle till late in the afternoon, when a new camp was formed, and we turned in early hoping for a good night's rest. But it was not to be. About twelve o'clock we were bidden saddle up, and hold a position near the Boer guns till daylight. None of us got a wink of sleep, as we were on guard. I was in charge of four horses linked together, and the poor half-starved brutes kept making for the grass, as they had had little to eat during the day. Now and again they would keep quiet for a few minutes, and then I tried to get a doze, but no sooner was I half asleep than they would make another rush and almost knock me over. With a start I sprang up, and as we were forbidden to speak above a whisper I muttered soft imprecations inwardly. However fond one may be of animals, a half starved horse under these circumstances can be very trying, especially when one is dead beat, having had no rest worth speaking of for three nights."

---

A few days later the horses, having probably been fed in the interim, stood the detachments in good stead, for they had to ride through the night to the Springfield Bridge. The writer continues: "We covered over thirty miles that day in heavy marching order, and didn't my spine make a fuss. But the scenery was so lovely it helped to keep us going. I have seen some beautiful spots in my time, but the views along the banks of the Tugela are beyond my humble description. Some of the hills are densely wooded on the Frere side with virgin bush, while in the open spaces aloe trees are very plentiful. The leaves of the latter make splendid firewood, and emit, when burning, rather a pungent but pleasant smell. The ash of this tree is used by the natives to mix with their snuff to give it a flavour. During the five days we were on the march we were on half rations and pretty nearly starved. We only had biscuits and Bully beef served out to us, and in none too great quantities. One day, however, we got a tin of cocoa and three mealies from a Kaffir, and on another the fare was composed of biscuits, tinned mutton, peaches, and Kaffir beer. This combination put a few of us *hors de combat*, especially as we made a 'square meal.' When the pangs of hunger assail us we draw our belts in two holes, and smoke enough to fill the vacancy for the time being. But we are now in Chieveley, in the land of plenty. The troops were awfully good to us last night, the men foregoing their beer so that we could get a drink all round, and even supplying us with some of their bread and going without themselves. The sight of bread quite upset me, and I polished off half a loaf in no time. To find ourselves eating fruit, bread, and groceries generally to our hearts' content after next door to no food seems very like a dream at first."

---

Someone cleverly summed up last Saturday and its doings as a day of "snow, sunshine, and shamrock." One might add, "particularly shamrock," for assuredly never has the little Irish emblem, or its substitutes, been seen in such profusion in England as upon St. Patrick's Day, 1900. The Queen's order that her Irish troops should wear a bit of green was obeyed not only by them, but by nearly all her subjects, English as well as Irish. Those whose purses were lightly filled had to be content with a diminutive sprig, for the demand in London far outran the supply, and consequently prices went up, but in the West End there was a striking display on the coats of both men and women. In Piccadilly we met a lady one side of whose jacket was quite green with her decoration, and I heard a rough young fellow behind me remark to his chum as he passed, "Looks as if she had got a good crop of mustard and cress a-coming up, don't it, Tom?" and I could not help laughing, the description was so accurate. Artificial shamrocks and bits of green ribbon were pressed into service when the real article failed, and anyhow the sentiment was equally genuine. There was no need of substitutes at the House of Commons, as numbers of boxes came to the Irish and other members from constituents, and at Windsor Castle, in addition to a large harp of shamrock sent Her Majesty by an unknown lady from Erin, the Royal table was a harmony in green, and the servants and officials all wore the Irish trefoil. The "Dunottar Castle," which took out Lady Roberts, and her two daughters, to join her husband, was decorated everywhere with flowers and shamrock, her private cabin—selected for her specially by her husband when he went out to South Africa on the same boat at Christmas-time—looking lovely. The sill of the port window was gay with Neapolitan violets, and on the table were large brass vases with posies of roses. The other cabin, arranged as a sitting-room, was festooned with smilax and hung with pink roses. In one corner stood a harp of shamrock tied with green ribbon, in another an offering of crimson roses and white lilac, whilst on the couch lay a large horseshoe of violets and lilies. The whole suggested quite a fairy bower. As Lady Roberts takes out six servants and an enormous amount of luggage, I do not fancy we shall see her back again until, all being well, she returns with her husband.

### Everybody's Opinion.

[Correspondence on all subjects is invited, but we cannot in any way be responsible for the opinions expressed by our correspondents. No communication can be entertained if the name and address of the correspondent is not given, as a guarantee of good faith but not necessarily for publication, or unless one side of the paper only is written on.]

### A CURIOUS CASE.

"Nurse Margaret" writes: I am engaged in nursing a case which began with influenza, followed by pneumonia and empyema. Patient is a girl of eleven; previous to illness she was an ordinary child, except that she was unusually reserved, but was obedient and tractable. Since her illness she has developed exactly opposite qualities, and has become untruthful, greedy, disobedient, and peevish. There does not seem to be anything in her physical condition to account for the change. I should be glad to know if any other nurses have had similar experience, and how long this mental and moral perversion is likely to last.

### A NEW SCHOOL FOR NURSES IN PARIS.

"A. H. M. D." writes: Allow me to give you a short answer to the question you put in your issue of March 3rd as to the practical training of the new Ecole Professionnelle des Infirmières à domicile. This nursing school has no footing in a hospital; the pupils are to be allowed to go to the municipal hospitals by leave of some of the visiting doctors, in their own wards and at the time of their daily visit, that is from nine a.m. to twelve o'clock. The civil hospitals of Paris possess a general nursing school for all of them, l'Ecole Municipale des Infirmiers et des Infirmières, opened since 1878. It is to be hoped that the new nursing school will soon be aware of how useless it is for its probationers to be at the hospital only at the time the wards are straight and patients quiet whilst doctors are sounding chests and teaching medical students. Nursing is still so backward in France that the very hospital doctors do not know what it all means, and think the best way to teach nurses their duties is to have them there at the time of their visit. The work done by the *infirmiers* and *infirmières* of the Paris hospitals does not deserve the name of nursing, and can only be a bad example for the probationers of the new nursing school.

### "THE RECORDING CLOCK."

"A Well-wisher of Hospitals" writes: I am a night nurse in a small hospital of three wards, which are, unfortunately, arranged rather far apart, so that it is not always easy to hear what takes place in the three at the same time. For some unknown reason to me, the committee of this hospital do not seem to place entire confidence in any of the night nurses they employ, and enforce the use of what is known, I believe, in asylums as a "recording clock." Now I consider the use of the clock also classes me among those who may not be trusted, although I am far too fond of my work to need a clock to keep me up to my duty. Each hour the clock must be marked in the three wards, and to do that it has to be banged into an iron case fixed in the wall, which is not accomplished without making a considerable noise. The latter, to my great annoyance, seldom fails to wake one or other of my patients. Those who know what nursing means can sympathise with me and realise how trying such an evil is to both nurses and patients. Now to the point. Is there not a remedy for the evil? Will not some inventive mind try and think of inventing a quieter kind of recording clock? I am not clever enough myself to think of anything suitable, and can only suggest that the iron cases into which the clocks are fitted should be lined with indiarubber linings, which might make some little difference. I have only taken night duty in the hospital mentioned for three months, so that I shall not benefit by any improvement that may happen to the recording clock, but I trust that others will. I am sure, however, that the use in small hospitals of recording clocks does not tend to raise the standard of the profession. Better not to employ nurses that require such reminders of duty.

### Presentations.

Allt-yr-yn Hospital, Newport, Mon.—On the retirement of Mrs. Inglis from the post of matron to the Allt-yr-yn Hospital, the Corporation, at their meeting in December, 1899, decided to record in their minutes their appreciation of her valuable services whilst acting as matron at the Fever Hospital. On the occasion of her marriage to Dr. R. J. Paton the members of the Sanitary Committee presented her with four fine etchings handsomely mounted and framed in oak, silver entrée dish, silver swivel tea-kettle, and a pair of silver mustard pots. The nursing staff presented her with two engravings of the late Lord Leighton's pictures, "Farewell" and "Hero's Last Watch." The domestic staff gave a pair of silver muffineers, silver carver rests, and handsome cushion.

Boys' Surgical Home, Benstead.—On resigning the charge of the Boys' Surgical Home, Benstead, Miss Chinnery was presented by the committee with a handsome travelling clock, with the following inscription: "Presented by the Committee to Miss Chinnery, in grateful appreciation of her work as Lady Superintendent of the Boys' Surgical Home, Benstead, 1897-1900." At the same time her staff nurse, Miss Gilling Lex, received a purse containing a cheque, accompanied by the good wishes of many friends.

### Minor Appointments.

Town's Hospital, Parliamentary Road, Glasgow.—Misses E. Mundie, J. M. Mortlock, E. Davies, W. Redmayne, M. Spencer, A. B. Thomson, C. F. Patrick, and J. Milne have been appointed Charge Nurses. Miss Mundie was trained at Her Majesty's Hospital, Stepney, and has since been nurse at the South Eastern Fever Hospital, London. Miss Mortlock was trained at the Coventry and Warwickshire Hospital, and has since been nurse at the New Hospital for Women, London. Miss Davies was trained at the Coventry and Warwickshire, and has since been nurse at the New Hospital for Women, London. Miss Redmayne was trained at Mile End Infirmary, and has subsequently been nurse at the South-Eastern Fever Hospital, London; the Hospital for Infectious Diseases, Havant; and the Haston Hospital, Tyne Dock, South Shields. Miss Spencer was trained at Barnhill Parochial Hospital, Glasgow, and has since been engaged in private nursing at Northampton. Misses Thomson, Patrick, and Milne were all trained at Samhill Parochial Hospital, Glasgow.

Cumberland Infirmary.—Miss F. E. Gibbon has been appointed Sister. She was trained for three years at the Liverpool Nurses' Training School connected with the Royal Infirmary. Since then she has been for one year on the private staff connected with the same institution, and has taken temporary duty as sister in surgical wards, and has been in charge of the operating theatre.

General Hospital, Canterbury.—Miss A. E. Lyall has been appointed Night Superintendent. She was trained at the Nightingale Home, and was staff nurse at St. Thomas's Hospital for three years. She was subsequently charge nurse in a diphtheria ward at the Fountain Fever Hospital, Tooting.

Royal Isle of Wight County Hospital, Ryde.—Miss Ada Kilby has been appointed Sister. She was trained at the Royal Exeter and Devon Hospital for three years, and has since held the appointment of staff nurse, theatre nurse, and holiday sister in the same hospital.

Beckett Hospital, Barnsley.—Miss Sarah Coney has been appointed Charge Nurse. She was trained at Luton Hospital, and has since been nurse at the Passmore Edwards Hospital, New Southgate, London.

Woolwich Union Infirmary.—Miss Maulda Johnson has been appointed Head Nurse. She was trained at Poplar and Stepney Sick Asylum for three years, and has since been chiefly engaged in private nursing.

Willesden Isolation Hospital.—Miss Bertha M. Duncombe has been appointed Charge Nurse. She was trained at he Willesden Isolation Hospital for three years.

### For Reading to the Sick.

YE have need of patience, that after ye have done the will of God ye might receive the promise.—*Heb.* x. 36.

Sweet Patience, come !
Not from a low and earthly source,
Waiting till things shall have their course ;
Not as accepting present pain
In hope of some hereafter gain ;
Not in a dull and sullen calm,
But in a breath of heavenly balm,
Bidding my weary heart submit
To bear whatever God sees fit.
Sweet Patience, come !
—*Hymns of the Church Militant.*

**Reading.**

The way of the Cross is not only the shortest way to Paradise, it is also the surest, because it is the one way by which our Father has pledged Himself to lead us thither. Happy they who have courage to go straight in this royal road, looking neither to the right hand nor to the left. All unworthy as you are, God has called you to become a saint by means of the Cross, and there have been moments when you could—so you felt—have accepted whatever trial it might please Him to send. Our Lord, entering upon His passion, cried out, "Father, the hour is come; glorify Thy Son." So do you say, "Lord, the hour of trial is come in which Thou willest to give Thy child a share in the Redeemer's glory; help me to do and bear all with a view to eternal life and glory."

Do you ever think how many temptations and faults are averted by the little crosses of daily life; how the graces of patience and forbearance and sympathy are fostered by the watchfulness they foster in you? How often bodily weakness and low spirits keep one from the mischiefs attendant on exuberant life and energy.

Lord, who has suffered all for me,
My peace and pardon to procure,
The lighter cross I bear for Thee,
Help me with patience to endure.—*Cowper.*

In all seasons of suffering—mental or bodily—it is a great help to try and dwell only upon the hour actually present. Bear or act for the best as in God's Sight for to-day, and leave to-morrow's cares absolutely to Him. They may never come in the shape you now imagine them ; or if they do they will bring with them their own peculiar strenthening grace, which does not attend their contemplation to-day. Remember the fable of the discontented pendulum, calculating how many million times it had to tick, and striking work in despair, until the wiser dial-plate reminded it that at no time more than a second actually exists, and it had only to tick once in each second. But do not be disheartened if one cross follows another. It does not rest with you to choose. Be sure that each cross exactly fits your need, is exactly that which to-day you need and can bear. If every day, every hope, every undertaking, seems to bring its cross; if there seems no outlet, no end, yet do not be disheartened. Let the cross but do its work in you, embrace it readily and cheerfully, and you will find the end, the outlet, perhaps just when you least looked for it, assuredly in Paradise.—*From Light of the Conscience.*

Let my soul beneath her load
Faint not, through the o'er-wearied flesh,
Let her hourly drink afresh
Love and peace from Thee, my God.
Let the body's pain and smart
Hinder not her flight to Thee
Nor the calm Thou givest me :
Keep Thou up the sinking heart.
*From the Lyra Germanica.*

### Notes and Queries.

THE Editor is always willing to answer in this column, without any fee, all reasonable questions, as soon as possible.

But the following rules must be carefully observed :—

1. Every communication must be accompanied by the name and address of the writer.
2. The question must always bear upon nursing, directly or indirectly.

If an answer is required by letter a fee of half-a-crown must be enclosed with the note containing the inquiry.

---

*Probationer* (38).

(238) Can you tell me of any hospitals or infirmaries where a probationer of 38 will be admitted?—*Y. W. C. A.*

So very few hospitals accept probationers after 35 years of age, that your best plan is to advertise.

*Nursing Instruction.*

(239) Would you kindly give me some information whether a married lady could get some instruction in trained nursing by going one or two evenings a week to help in the wards of the infirmary, and what the terms would be, also could she attend the lectures to probationers?—*Violet.*

You could do no harm by applying to the matron of your nearest infirmary, but it is not likely that she will admit outside students.

*Hemiplegia.*

(240) Can you tell me of any institution which would provide for a nurse with hemiplegia? She is about 43 years of age and entirely without means. The attack occurred when on duty, and at present she has slight aphasia, complete paralysis of right arm, and only very limited movement in the leg.—*London.*

There is no institution affording an asylum for sick nurses especially.

*Furniture.*

(241) Can you tell me any firm where I can purchase small combination furniture suitable for nurses' cubicles?—*C. U. W. M.*

Many of the furnishing warehouses make special terms for public institutions. The plan usually pursued is to ask more than one local firm to send in estimates for the article required. Why not have fixtures?

*Death-rate Amongst Nurses.*

(242) 1. Is the nursing profession considered in England to have a higher death-rate than other professions? 2. Do the matrons, sisters, and staff nurses get any special pay for teaching probationers in hospitals having good nursing schools attached?—*A. H. M. D.*

The death-rate amongst nurses is certainly higher. 2. No; a matron's, &c., salaries cover all her duties, including the training of probationers.

*Ambulance Classes.*

(243) Will you kindly tell me where to apply to join an ambulance class, and what is the fee? I am anxious to become a nurse, and think it might be useful.—*C. E. L.*

Apply to the secretary, St. John Ambulance Association, St. John's Gate, Clerkenwell. It would, however, be better to join classes on cookery and theoretical nursing subjects, for, as a rule, previous practical training does not recommend a probationer to matrons.

*Poison and Antidotes.*

(244) Could you please tell me the name of a book giving the distinctive symptoms of poisoning by various drugs, &c., with remedies?—*K. S. L.*

"What to Do in Cases of Poisoning," by Murrell; price 3s. 6d. Any bookseller would procure it for you.

*Uniform.*

(245) I have been nurse in this institution for two years, and charge nurse for the last fourteen months. I received my indoor and outdoor uniform ten months ago, and, as I am leaving, I should like to know if I must return it to the hospital or not. Could I claim it on the completion of twelve months?—*R. C.*

The possession of the uniform depends upon the terms of your agreement. As a rule the uniform belongs to the institution, and the nurse has the use of it during her period of service only.

*Child's Nurse.*

(246) Can you give me the address of the training home for children's nurses?—*H. L. H.*

The Norland Institute for Training Children's Nurses, 29, Holland Park Avenue, Notting Hill, W.

*Book.*

(247) Can you recommend me a reliable, but not expensive, book on the treatment of skin diseases, psoriasis particularly?—*Inquirer.*

"Diseases of the Skin; their Description, Pathology, Diagnosis, and Treatment," by H. R. Crocker, 24s.; "On Psoriasis or Lepra," by George Gaskoin, 5s., may be referred to, but "treatment" is beyond a nurse's function.

*Standard Books of Reference.*

"The Nursing Profession: How and Where to Train." 2s. net.
"The Nurses' Dictionary of Medical Terms." 2s. 6d. net.
"Burdett's Series of Nursing Text-Books." 1s. each.
"A Handbook for Nurses." (Illustrated.) 5s.
"Nursing: Its Theory and Practice." New Edition. 3s. 6d.
"Helps in Sickness and to Health." Fifteenth Thousand. 5s.

All these are published by THE SCIENTIFIC PRESS, Ltd., and may be obtained through any bookseller or direct from the publishers, 28 & 29, Southampton Street, London, W.C.

